# Inhibition of PI3K-AKT-mTOR pathway sensitizes endometrial cancer cell lines to PARP inhibitors

**DOI:** 10.1186/s12885-017-3639-0

**Published:** 2017-09-08

**Authors:** Charles-André Philip, Ido Laskov, Marie-Claude Beauchamp, Maud Marques, Oreekha Amin, Joanna Bitharas, Roy Kessous, Liron Kogan, Tahira Baloch, Walter H. Gotlieb, Amber Yasmeen

**Affiliations:** 1 Division of Gynecologic Oncology, Jewish General Hospital, McGill University, Montreal, QC Canada; 2 Segal Cancer Center, Lady Davis Institute of Medical Research, McGill University, 3755 Cote Ste. Catherine Road, Montreal, QC H3T 1E2 Canada; 3 Department of Oncology, McGill University, Montreal, QC Canada; 4 Department of Obstetrics and Gynecology, Lis Maternity Hospital, Tel Aviv Sourasky Medical Center, Tel Aviv University, Tel Aviv, Israel

**Keywords:** Endometrial cancer, PTEN, PI3K/mTOR pathway, PARP inhibitor, DNA repair pathway, RAD51

## Abstract

**Background:**

Phosphatase and Tensin homolog (PTEN) is a tumor suppressor gene. Loss of its function is the most frequent genetic alteration in endometrioid endometrial cancers (70–80%) and high grade tumors (90%). We assessed the sensitivity of endometrial cancer cell lines to PARP inhibitors (olaparib and BMN-673) and a PI3K inhibitor (BKM-120), alone or in combination, in the context of their PTEN mutation status. We also highlighted a direct pathway linking PTEN to DNA repair.

**Methods:**

Using endometrial cancer cellular models with known PTEN status, we evaluated their homologous recombination (HR) functionality by RAD51 foci formation assay. The 50% Inhibitory concentration (IC50) of PI3K and PARP inhibitors in these cells was assessed, and western blotting was performed to determine the expression of proteins involved in the PI3K/mTOR pathway. Moreover, we explored the interaction between RAD51 and PI3K/mTOR by immunofluorescence. Next, the combination effect of PI3K and PARP inhibitors on cell proliferation was evaluated by a clonogenic assay.

**Results:**

Cells with mutated PTEN showed over-activation of the PI3K/mTOR pathway. These cells were more sensitive to PARP inhibition compared to PTEN wild-type cells. In addition, PI3K inhibitor treatment reduced RAD51 foci formation in PTEN mutated cells, and sensitized these cells to PARP inhibitor.

**Conclusion:**

Targeting both PARP and PI3K might lead to improved personalized therapeutic approaches in endometrial cancer patients with PTEN mutations. Understanding the complex interaction of PTEN mutations with DNA repair in endometrial cancer will help to better select patients that are likely to respond to some of the new and costly targeted therapies.

## Background

Endometrial cancer is the most common gynecologic cancer in developed countries [1], and its incidence is increasing [2]. Over 50% of women with endometrial carcinoma present with early-stage, low-risk disease, and are treated by surgery alone [3]. Adjuvant therapy recommendations are based on the individual patient’s risk of disease recurrence using clinicopathologic factors such as age, stage, histologic subtype, tumor grade, and lymphovascular space invasion (LVSI) [4]. Clinical and pathologic risk stratification is limited and many patients are under- or over treated as a result [5]. Risk assessment might be improved by integrating molecular biomarkers predictive of an individual tumor behavior. In addition, a better understanding of the different molecular mechanisms of endometrial cancer subtypes could provide insight into the development of improved targeted therapeutic strategies [5, 6].

Phosphatase and Tensin homolog (PTEN) is a tumor suppressor gene located on chromosome 10q23 and responsible for a dual specific tyrosine phosphatase activity [7, 8]. The main known role of PTEN is the down regulation of the PI3K-AKT pathway, through the dephosphorylation of Phosphatidylinositol [3–5]-trisphosphate (PIP3) to PIP2 which antagonizes the activity of PI3K. As PIP3 is essential to the phosphorylation of AKT by the Phosphoinositide-dependent kinase-1 (PDK-1), PTEN leads to the inhibition of the Phosphoinositide 3-kinase (PI3K)-AKT-mammalian Target of Rapamycin (mTOR) pathway [9, 10]. Since this pathway is responsible for several cellular activities including inhibition of apoptosis, PTEN loss-of-function is frequently implicated in oncogenesis [9].

Loss-of-function mutations in PTEN gene are the most frequent genetic alterations in endometrioid endometrial cancer (70 – 80%) and in up to 90% of high grade tumors [11, 12]. PTEN mutations are associated with an activation of the PI3K-AKT-mTOR pathway and recent publications have linked PTEN and the PI3K-AKT-mTOR pathway to the mechanism of DNA double strand break (DSB) repair through homologous recombination [13, 14].

Poly ADP-ribose polymerase-1 (PARP-1) is an important protein involved in DNA single strand break (SSB) repair [15]. A dysfunction of SSB repair leads to an accumulation of DSB’s, which are then repaired by one of the DSB repair mechanisms, the most important one being the DNA homologous recombination (HR) repair pathway. Therefore, following DNA damage through single-strand breaks, inhibition of PARP-1 leads to accumulation of DSB’s in HR-deficient cells, such as BRCA1/2-mutated cells. This effect is toxic and induces apoptosis [16–20]. This mechanism of targeted therapy has been named synthetic lethality. Olaparib (AZD-2281, AstraZeneca®) is currently the only PARP-inhibitor approved for clinical use by both the Federal Drug Agency (FDA) and the European Medicines Agency (EMA) in ovarian cancer. Talazoparib (BMN-673, AdooQ Bioscience) is a new PARP-inhibitor that has shown promising results in both breast and endometrial cancers in vitro [21, 22]. While PARP inhibitors are only approved clinically in patients with mutations in BRCA1 or BRCA2, studies have repeatedly shown that cells with defects in other HR genes might also be sensitive to PARP inhibitors [23]. It has been previously shown that PTEN loss-of-function is associated with higher sensitivity to PARP inhibitors through a synthetic lethal mechanism in prostate, lung, cerebral, [24–28] and, notably, endometrial cancer [29, 30]. However, the later observation is challenged by another study showing that some PTEN-mutated endometrial cancer cell lines were not sensitive to olaparib [31]. The reasons for this lack of sensitivity, and whether other agents might sensitize resistant cells to PARP inhibitors, remain unclear.

BKM-120 (Novartis), a Pan-PI3K inhibitor, inhibits PI3K isoforms with a 50-fold selectively over other protein kinases [32]. In the first clinical trials, BKM-120 use as a single agent and in combination with other forms of treatment showed promising antitumor activity with acceptable and limited adverse effects [33–36]. This drug was also efficient in reducing tumor volume in primary xenograft model with PI3K/AKT activated endometrial cancer [37]. BKM-120-mediated PI3K inhibition was shown to impair BRCA1/−2 mRNA and protein expression, which indicates that it may be an efficacious inhibitor of HR DNA repair functionality. In the same study, it sensitized wild-type BRCA, triple-negative breast cancer cell lines to PARP inhibition [14].

Successful treatment using PARP inhibition depends on HR functionality. While PARP inhibitors are only approved in BRCA1/2 mutations, PTEN mutations and P13K inhibition have been proposed as alternative inducers of HR functionality.

Our goal in the present study was to clarify whether PTEN mutations mediated the inhibitory efficiency of PARP-inhibitors (olaparib and BMN-673) through HR functionality, and evaluate whether the Pan-PI3K inhibitor (BKM-120) sensitized endometrial cancer cell lines to PARP inhibitors through HR suppression. Even though many women with endometrial cancer are cured, those with recurrent disease certainly need more treatment options. Results from this paper may provide an additional step towards developing other treatment options in women with recurrent endometrial cancer.

## Methods

### Cells lines

Four endometrial cancer cell lines were used in this study (Table 1). Two cell lines were PTEN mutated and two were PTEN wild-type; all were previously purchased and kindly gifted by Dr. Jennifer K. Richer (Anschutz Medical Campus, University of Colorado, USA). HEC-50 PTEN wild-type is a High Grade (grade 3), type II, non-endometrioid endometrial adenocarcinoma cell line derived from ascitic fluid of a patient with recurrence [38, 39] and showed the capacity to differentiate into a papillary serous phenotype in a mouse model [40]. HEC-1B is a PTEN wild-type cell line that has been derived from a localized high grade endometrioid adenocarcinoma [41]. Ishikawa is a moderately differentiated (grade 2) endometrioid endometrial cancer expressing estrogen receptor and is PTEN mutated [Codon 289 del 1 bp (A) and Codons 317–318 del 4 bp (ACTT)]. These deletions are responsible for a truncated and non-functional protein, or the degradation of PTEN [42–44]. AN3CA is a high grade endometrioid endometrial cancer cell line derived from a lymph node metastasis displaying a PTEN mutation (homozygote for codon 130 del 1 bp (G)) [43–46]. All the cells lines were authenticated by short tandem repeat (STR) profiling by the DNA sequencing and analysis core of the University of Colorado which has extensive experience in evaluation of gynecological cell lines [47]. A minimum of 85% of similarity between a reference profile and our cell lines was observed. AN3CA, HEC-1B and HEC-50 were cultured in Eagle’s Minimum Essential Medium (EMEM) associated to 10% Foetal bovine serum (FBS) and 0.02 mg/mL gentamycin. Ishikawa cells were cultured in EMEM +2 mM Glutamine +1% Non-essential Amino Acids (NEAA) + 5% FBS + 0.02 mg/mL gentamycin [48]. Each cell line was passage every 4 – 6 days. All cells were maintained at 37 °C in a 5% CO2, 95% air atmosphere incubator. All assays were performed in the respective cell medium.

**Table 1:** Clinical characteristics with PTEN status of cell lines examined

Cell line	Tumor histology	Grade	Recurrent/ metastatic	PTEN status	Mutation type	Specific mutations	Protein expression	References
HEC-50	Non-endometrioid	3	+	Wild type	–	–	+	38–40
HEC-1B	Endometrioid	3	–	Wild type	–	–	+	41
ISHIKAWA	Endometrioid	2	–	Mutated	Frameshift	Codon 289del A Codons 317–318 del ACTT	–	42–44
AN3CA	Endometrioid	3	+	Mutated	Nonsense	Codon 130 del G	–	43–46

### PARP-1 and PI3K inhibitors

Olaparib (AZD2281), Talazoparib (BMN-673) and BKM-120 (NVP-BKM120) were ordered from AdooQ Bioscience (Catalog number #A10111, #A11243 and #A11016 respectively) diluted in 10 mM stocks in DMSO and stored at −20 °C. During experiments, aliquots of 1000-fold the final concentration were prepared in DMSO for each concentration used and stored at −20 °C. New aliquots were prepared directly from stocks every 5 – 10 uses to minimize drug degradation. Drug concentrations were designed according to the available clinical trials literature. As reported in a phase 1 clinical trial, the maximal plasma concentration of olaparib was between 3 and 8 μg/mL, which correspond to 6.9–18 μM [49]. Thus, the concentrations used in the present study ranged between 0.01 – 10 μM of olaparib which is at the lower range of that used in the clinical trial. Similarly, the maximal plasma concentration of BKM-120 was reported to be between 500 – 1500 ng/mL, corresponding to 1.2–3.7 μM [50]. Accordingly, we have used overlapping concentrations ranging from 0.1 to 5 μM of BKM-120 in our in vitro assays. With regard to Talazoparib (BMN-673), since there is still no clinical trial reporting its plasmatic concentration, its inhibitory activity was first tested in similar range of concentrations to that employed for olaparib. Following our preliminary results, we realized that Talazoparib had a smaller inhibitory dose than olaparib and modified the dosage accordingly.

### Clonogenic and cell proliferation assays

Four hundred to eight hundred cells were plated in 6-well in duplicates. Cells were washed and fresh medium was added in the presence or absence of increasing doses of PI3K- inhibitor (BKM-120) and PARP-inhibitor (olaparib or BMN-673) alone and in combination after 24 h. Media containing the drug was refreshed on day 3. DMSO was used as a control. The experiment was discontinued when the clones reached 50 cells/clone in the DMSO-vector wells (7 to 12 days) and colonies were fixed and stained with 1.5 ml of 6.0% glutaraldehyde and 0.5% crystal violet and colonies were counted using the GelCount, (Oxford optronix, UK). The surviving fraction (SF) of cells was calculated as follows: SF = $$ \frac{Number of colonies formed after treatment\ }{Number of cells seeded\ x\  Plating Efficiency\ } $$, where Plating Efficiency = $$ \frac{Number of colonies formed in control}{Number of cells seeded} $$ [51]. Chou and Talalay method was used to assess the interaction between two inhibitors [52]. This method quantitatively describes the interaction between two or more drugs, with combination index (CI) values less than 1 indicating synergistic interactions, values greater than 1 indicate antagonistic interactions, and values equal to 1 indicate additive interactions. Calculations of the CI values were performed with CompuSyn Software (ComboSyn, Inc., Paramus, NJ. 07652 USA).

Proliferation assays were used to determine the inhibitory effect of drugs on the studied cell lines. Control plates were created for each cell line using 6 wells of a 24-wells plate. Ten thousand cells in 1 mL were plated in 24 well plates for drug assessment. After 24 h of standard culture at 37 °C (D0), control plates were fixed using a 4% paraformaldehyde (PFA) solution for 30 min and then stored in 0.4% PFA at 4 °C. At the same time, plates were treated with olaparib (0.01 μM, 0.1 μM, 1 μM, 5 μM and 10 μM) and BKM-120 (0.1 μM, 0.5 μM, 1 μM, 2.5 μM, 5 μM). Each concentration was tested in triplicate. DMSO was used as control. Cells were fixed using a similar procedure at day 3 (D3) and 5 (D5). All drugs and vector-controls were refreshed at Day 3. After removal of PFA, a 0.1% crystal violet/10% Ethanol solution was used to stain the fixed cells and quantify proliferation (250 μL per well during 30 min at room temperature with shaking). The wells were then aspirated and allowed to air-dry at least 2 h. A 10% acetic acid was used to dissolve the staining dye (500 μL/well). At least, the 200 μL of each well were transferred into a 96-wells plate, before the absorbance was measured at 590 nm by spectrophotometry, as it is assumed that the level of absorbance is proportional to the number of cells in the well at the time of the fixation.

### Protein extraction and western blot analysis

Cells were harvested (2 mL 0.25% Trypsin-EDTA 1×, Wisen Bio Products) and then lysed in 500 μL of radioimmunoprecipitation assay (RIPA) buffer (25 mM/L Tris-HCl pH 7.6, 150 mM/L NaCl, 1% NP-40, 1% sodium deoxycholate, 0.1% SDS and 1 mM/L EDTA). Protein concentration was determined using bicinchoninic acid assay (BCA) kit (Ref 23,227, Pierce) using a spectrophotometer at 570 nm. Protein lysates (10–25 μg) were separated electrophoretically on a 7.5 – 12% denaturing SDS-polyacrylamide gels and transferred to 0.2 μm nitrocellulose membranes. Primary antibodies specific for PTEN (#9552; Cell Signaling, Beverly, MA, USA. 1:1000), PI3K (#4238; Cell Signaling; 1:500), phospho-PI3K (#4284; Cell Signaling; 1:500), AKT (#9272; Cell Signaling; 1:1000), phospho-AKT (Ser473, #9271S; Cell Signaling; 1:1000), S6 Ribosomal Protein (#2217; Cell Signaling; 1:1000), phospho-S6 (Ser240/244, #2215; Cell Signaling; 1:1000), and β-actin (#4967, Cell Signaling; 1:2000) were diluted in 0.1% Tween-PBS/5% Milk and put in presence of the membrane overnight at 4 °C. After 3 washing (0.1%Tween-PBS1X), membranes were exposed to secondary anti-rabbit-horseradish peroxidase (HRP; L170–6515; Bio-Rad, USA; 1:10,000) or anti-mouse HRP (L170–6516; Bio-Rad; 1:10,000) for 1 h at room temperature. Immunoreactive proteins were detected by chemiluminescence (WBKLS0500; Immobilon Western, Millipore) and autoradiography [53].

### Gene silencing and transient transfection

PTEN specific small hairpin RNA (shRNA) containing the following sequence: CCGGCCACAAATGAAGGGATATAAACTCGAGTTTATATCCCTTCATTTGTGGTTTTT were ordered in Bacterial Glycerol Stock (#TRCN0000002749, Sigma-Aldrich, Saint-Louis, MO, USA). shRNA were annealed 4 min at 95 °C in a PCR machine, inserted into pLKO.1 cloning vector (gift from Bob Weinberg, Addgene plasmid # 8453) and amplified in DH5-alpha bacterial cells before antibiotic selection by 100 μg/mL of ampicillin. PTEN wild type cell lines (HEC-50 and HEC-1B) were plated at approximately 30% confluence in 100-mm plates and incubated for 24 h before transfection with 2 μg of pLKO.1 PTEN shRNA plasmid. An empty plasmid was used as a control (pLKO.1 puro, Addgene plasmid #8453). Successfully transfected cells were selected using puromycin [20].

### Immunofluorescence analysis

1 × 10^5^ cells / well were seeded in 6-well plates on a sterile cover slip. After Twenty-four hours, when cells reached ~60% confluency, then cells were washed and the medium was replaced with medium containing 500 nM doxorubicin for 1 h and allowed to recover for ~6 h. In another setting, the cells were treated with medium containing 1 μM BKM-120 for 24 h, followed by 500 nM doxorubicin for 1 h and allowed to recover for ~6 h. Fixation, permeablization and antibodies staining were performed as described earlier [20]. Images were analyzed and quantified using ImageJ software (NIH) [20].

### Statistical analysis

Data are expressed as the means ± standard deviations of three independent determinations. The significance of differences between the two samples was analyzed using Student’s t-test, and a *p*-value of <0.05 was considered statistically significant.

## Results

### Determination of PTEN protein expression and PI3K-AKT pathway activation in endometrial cancer cell lines

**Fig. 1:**
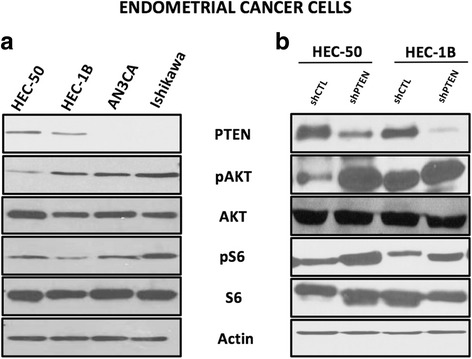
PTEN expression and PI3K-AKT-mTOR pathway activation in endometrial cancer cells. **a** In 12% SDS gel western blot using protein extracts collected in standard conditions (Media containing 10% FBS), HEC-50 and HEC-1B cells expressed PTEN protein, while AN3CA and Ishikawa did not. As expected with such a mutation, Ishikawa had high level of phospho-AKT (p-AKT) and phospho-S6 (p-S6). HEC-50, displayed a lower level of p-AKT despite a PTEN mutation. **b** Reduced expression of PTEN was observed in HEC-50 and HEC-1B transfected with shPTEN. Results shown are one representative experiment out of three independent experiments

We evaluated PTEN protein expression in endometrial cancer cells, as shown in Fig. 1a. Western blot analysis confirmed that HEC-50 and HEC-1B cells were PTEN wild-type, whereas Ishikawa and AN3CA cells carrying PTEN deletion mutations (resulting in PTEN truncated or degraded proteins), did not express wild type PTEN protein, confirming previously published data about their PTEN mutated status [43, 54]. In standard culture condition, Ishikawa had high levels of phospho-AKT (p-AKT) and phospho-S6 (p-S6), thus, as expected from PTEN mutated cell lines, it is expressing an activated PI3K-AKT-mTOR pathway. Conversely, AN3CA displayed a lower level of p-AKT but a high level of p-S6. HEC-50 also had a lower level of p-AKT but p-S6 was present similarly to the PTEN mutated cell lines. Therefore, to confirm the effect of PTEN on pAKT and pS6 expression level, HEC-50 and HEC-1B cells were transfected with shPTEN and were assessed by western blot. As shown in Fig. 1b, we found reduced levels of PTEN protein as well as increased p-AKT and p-S6 in the PTEN-depleted cells compared to the control cells.

### Absence of PTEN sensitize endometrial cancer cells to PARP inhibitors

**Fig. 2:**
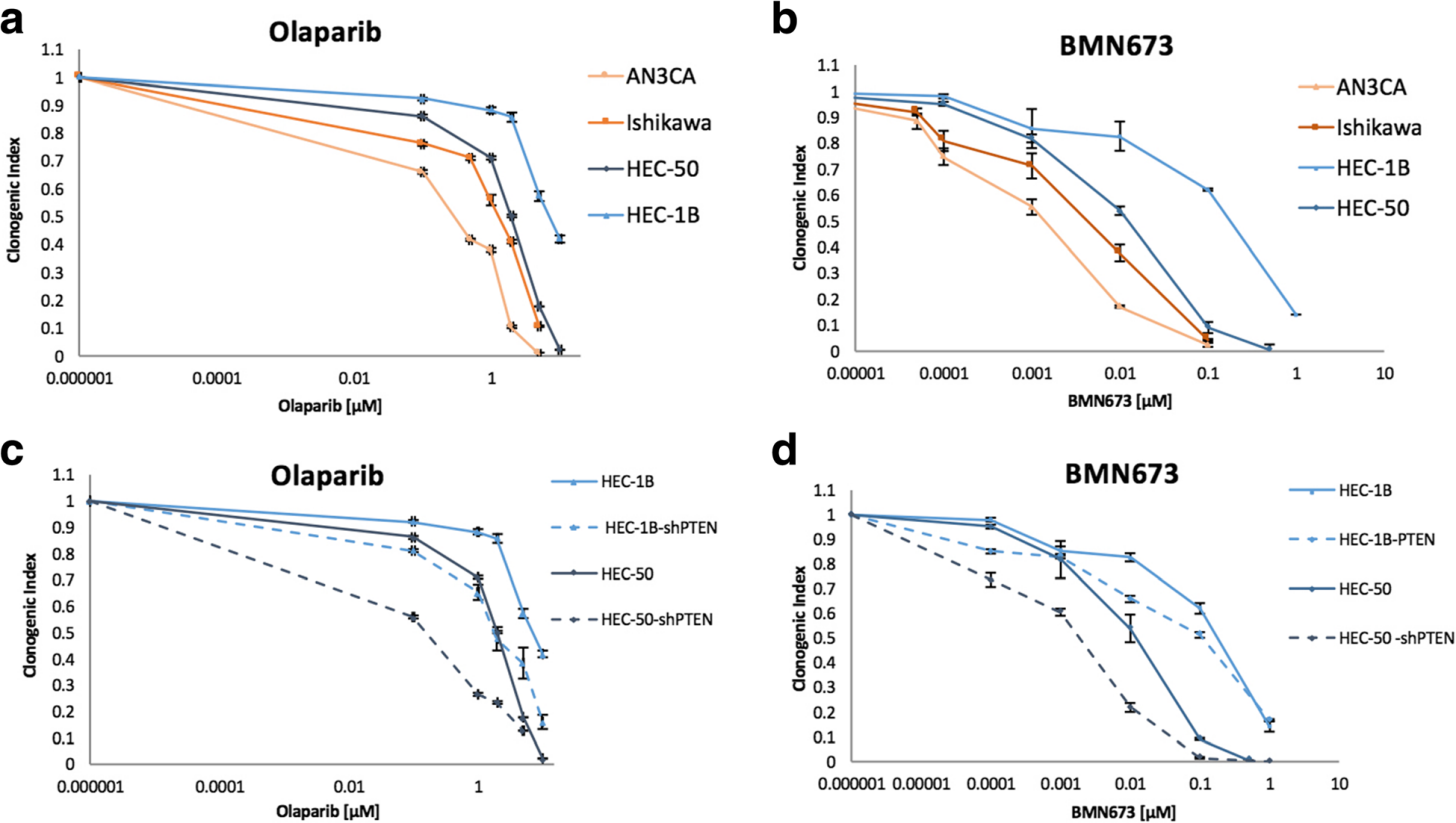
PTEN mutations sensitize endometrial cancer cells to PARP inhibitors. In a clonogenic assay at day 5 (D5), the sensitivity to olaparib is higher for the PTEN mutated cell lines, Ishikawa and AN3CA compared to the PTEN wild-type cell lines, HEC-50 and HEC-B1 (**a**). BMN-673 shows a higher efficiency of inhibitory activity than olaparib in all cell lines (**b**). The transfected shPTEN cell lines were more sensitive to both olaparib and BMN-637 compared to their wild type PTEN counterparts **c** and **d** which confirms the higher sensitivity to PARP-inhibitors when PTEN is mutated

We next evaluated the sensitivity of the endometrial cancer cells to PARP-inhibitors by clonogenic and cell proliferation assays. We observed that the mutated cell lines (AN3CA and Ishikawa) were more sensitive to the PARP-inhibitor olaparib compared to the PTEN wild-type cell lines (HEC-50 and HEC-1B) after 5 days (Fig. 2a). Similar data was observed after 3 days (data not shown). The median inhibitory concentration (IC50) was 0.5 μM for AN3CA cells and 3 μM for ishikawa cells compared to 6 μM for HEC-50 cells and 10 μM for HEC-1B cells. The same pattern was observed with a more potent PARP-inhibitor, BMN-673 (Fig. 2b), with IC50 at 0.005 μM for AN3CA cells and 0.008 μM for ishikawa cells. To confirm the difference in sensitivity to PARP-inhibitors, between the PTEN mutated to the PTEN non-mutated cells, we transfected the PTEN wild-type cells, HEC-50 and HEC-1B with shPTEN. As shown in Fig. 2c-d and Table 2, the transfected shPTEN cells were more sensitive to both olaparib and BMN-673 compared to their un-transfected counterparts. A proliferation assay showed overall similar results (data not shown).

**Table 2:** Summary of the median inhibitory concentrations (IC50) of Olaparib and BMN-673 inhibitors on the endometrial cancer cell lines by clonogenic assay

Cell line	Inhibitor	IC50(μM)	Inhibitor	IC50 (μM)
AN3CA	Olaparib	0.5	BMN673	0.001
Ishikawa	Olaparib	3	BMN673	0.003
HEC-50	Olaparib	6	BMN673	0.005
HEC-50 shPTEN	Olaparib	0.1	BMN673	0.004
HEC-1B	Olaparib	10	BMN673	0.125
HEC-1B shPTEN	Olaparib	2.1	BMN673	0.051

### PTEN mutation increases the sensitivity of a PARP-inhibitor and a PI3K-inhibitor in combination

**Fig. 3:**
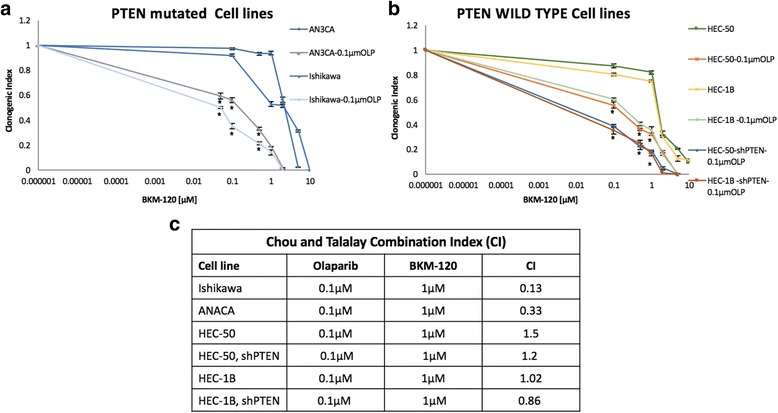
BKM-120 and olaparib in combination effect in PTEN wild-type and mutated endometrial cancer cells. PTEN mutated **a** and wild-type **b** cancer cells were treated with increasing doses of BKM-120 in the presence of low doses of 0.1 μM or 0.5 μM olaparib for 7 days and their effect on cell proliferation was determined using the clonogenic assay. **c** The combination index was calculated where CI < 1 indicates synergy between the two drugs. Data represents the average of three independent experiments. Data not shown for 0.5 μM olaparib.*p value <0.05

We evaluated whether HR impairment provoked by PI3K inhibition conferred increased sensitivity to PARP inhibition in the context of PTEN mutation. We cultured these endometrial cancer cell lines in a fixed, low dose of olaparib (0.1 μM and 0.5 μM) and increasing drug concentrations of BKM-120 ranging from 0 to 10 μM. The idea was to mix a sublethal dose of olaparib (number of growing clones comprised between 0.8 – 1.2 fold the control number), with increasing concentrations of BKM-120 to see if it was enough to induce a stronger inhibitory effect on cell growth compared to that of BKM-120 by itself. As shown in Fig. 3a. we found a significant decrease in the clonogenic indices of PTEN-mutated cells with combination treatment as compared to PI3K and PARP-inhibitor alone. Further, to determine if the PI3K and PARP-inhibitors combination were additive or synergistic, we used the ‘multiple drug effect analysis’ method of Chou and Talalay (see Materials and Methods) [52]. Interestingly, in both PTEN mutated cell lines tested, we observed the combination treatment to be strongly synergistic, with a CI ˂1.0 (Fig. 3c). When we looked at the PTEN wild-type cells, we also found decreased survival with combination treatment as compared to PI3K and PARP-inhibitor alone (Fig. 3b). However, in both wild type cell lines tested, we observed the CI to be close to 1, suggesting an additive effect (Fig. 3c). The CI for HEC-50 was 1.5 (olaparib 0.1 μM and BKM-120 1 μM) and 1.02 for HEC-1B (olaparib 0.1 μM and BKM-120 1 μM). This effect was less pronounced in shPTEN transfected cells, with somewhat lower CI: 1.2 for HEC-50, shPTEN (olaparib 0.1 μM and BKM-120 1 μM) and 0.86 for HEC-1B, shPTEN (olaparib 0.1 μM and BKM-120 1 μM).

### Decreased homologous recombination (HR) functionality in PTEN mutated endometrial cancer cells

**Fig. 4:**
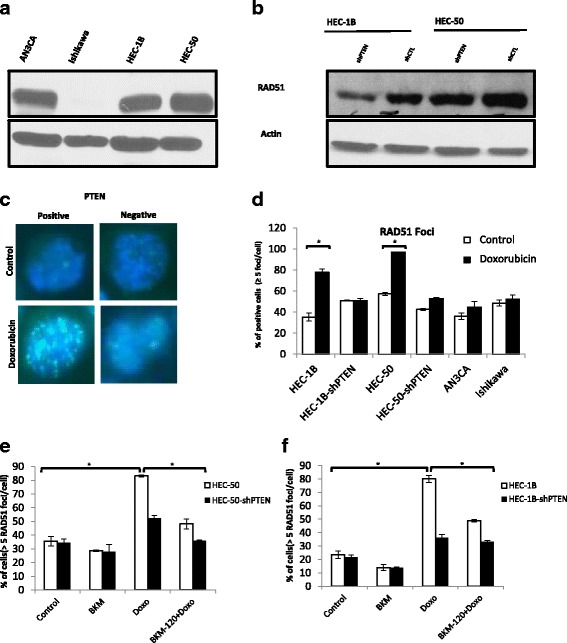
Reduced RAD51 foci formation in PTEN mutated endometrial cancer cells. Baseline RAD51 protein expression in the endometrial cancer cells (**a** and **b**). Cells were treated with 500 nM of Doxorubicin for 1 h, allowed to recover for 6 h and then fixed for immunofluorescence. Immunofluorescence staining images of RAD51 foci in nuclei of endometrial cancer cells with respect to the PTEN protein expression are shown at 100X magnification (**c**). Quantitative representation of the percentage of cells with positive RAD51 foci is shown in fig (**d**). PI3K inhibition decreases doxorubicin-induced RAD51 foci formation in these cells (**e** and **f**). Cells with >5 foci/nucleus were considered positive. Results represent the average of three independent experiments. *p < 0.05

We then assessed the correlation of PTEN protein expression and HR functionality in these cells. To assess HR functionality, we evaluated the baseline levels of RAD51 protein expression and foci formation in the PTEN-mutated and wild-type endometrial cancer cells upon DNA damage, with 1 h treatment of 500 nM doxorubicin, by immunofluorescence, as an indication of the cells ability to repair DNA double strand break [51]. As shown in Fig. 4a only one of the two PTEN mutated cells (Ishikawa) did not express RAD51 protein at all. In addition, the two shPTEN transfected wild type cells expressed a decreased amount of RAD51 (Fig.4b). As shown in Fig. 4c and d, we observed a significant reduction in nuclear RAD51 foci formation after doxorubicin treatment in cells having low or absent PTEN protein expression, suggesting a lower capacity of DNA repair through HR. Next, using RAD51 foci formation assay, we found that the cells treated with the 1 μM PI3K- inhibitor (BKM-120), showed decreased formation of RAD51 foci after induction of DNA damage with 500 nM doxorubicin treatment, as compared to cells treated with 500 nM doxorubicin treatment alone (Fig. 4e and f). This reduction was probably due to a decrease in RAD51 protein levels in response to treatment with the PI3K-inhibitor (data not shown) suggesting that PI3K inhibition directly impacts HR functionality in our studied cancer cells.

## Discussion

PTEN loss-of-function is the most frequent genetic alteration in endometrioid endometrial cancer reaching up to 90% frequency in high grade tumors [11, 12]. Those mutations are usually associated with an activation of PI3K-AKT-mTOR pathway, as PTEN role is to down-regulate this pathway through the dephosphorylation of PIP3 [55]. Our present data indicates that PTEN provides negative feedback to the PI3K-AKT-mTOR pathway, as demonstrated by the enhanced protein levels of p-AKT and p-S6 in endometrial cancer cells having loss-of-function mutations of PTEN (Fig. 1a and b). Previous studies demonstrate that over activation of AKT is responsible for several cellular dysregulations such as apoptosis inhibition, and activation of proliferation and glycolysis through the serine-threonine phosphorylation of many substrate proteins including mTORC1 complex [56]. The hyperactivation of the PI3K-AKT-mTOR pathway, is implicated in the oncogenic transformation of many tumors and in particular in endometrial cancer with PTEN loss-of-function [9]. Therefore, an attempt to inhibit this pathway seems to be of clinical relevance [57].

The link between PTEN mutation and PARP inhibitor sensitivity has been described in several cancers. Using manipulated human colon cancer cell lines, Mendes-Pereira et al. have shown that PTEN−/− cell lines were 20 times more sensitive to olaparib than their wild-type counterpart [24]. Similar results were also obtained with velaparib in glioblastoma cell lines [27], with rucaparib in prostate cancer [26] and with olaparib in lung cancer cell lines [28]. In endometrial cancer, there is also a publication concerning a higher sensitivity to olaparib in PTEN mutated cells in low oestrogen concentration condition [58]. Dedes et al. reported that KU0058948, a first generation PARP-inhibitor, had higher efficiency in PTEN-deficient endometrial cells than in wild-type PTEN endometrial carcinoma cell lines [29]. Finally, Forster et al. reported on women presenting with a cerebral metastatic PTEN mutated endometrial cancer that have clinically responded to olaparib, followed by a 10-month survival [30]. On the other hand, Miasaka et al. recently published that olaparib was effective on certain endometrial cancer cell lines, but that inactivation of PTEN was not in correlation to the DNA repair function [43]. In our present study, we found that PARP inhibitors seem to be more efficient in suppressing the growth of PTEN mutated cell lines and that BMN-673 is more potent inhibitor than olaparib (Fig. 2). This is in concordance to previous reports, showing that BMN-673 is superior to other PARP inhibitors in BRCA and PTEN mutated cell lines of various cancers [22].

There is some evidence linking PI3K-AKT-mTOR pathway to DNA damage response (DDR) which could explain why loss of expression of PTEN is associated to a higher sensitivity to PARP inhibitors. PI3K might contribute to DSB repair by interacting with the HR complex [13]. Similarly, the suppression of PI3K function has been shown to impair HR [14]. PI3K seems also to play a critical role in RAD51 recruitment in response to DNA damage [59]. Thus, activation of the PI3K-AKT-mTOR pathway may be associated with a higher level of DDR and should lead to a higher resistance to PARP inhibitors. This association has been suggested in a recent study using BMN-673 in lung cancer [60].

Since PI3K-AKT-mTOR activation is responsible for an increase in cell survival, cell proliferation and, is associated with an activation of DNA repair, we speculated that the combination of PARP and PI3K-inhibitors could be beneficial in endometrial cancer.

We found, that the combination of olaparib and BKM-120 is more efficient in suppressing the growth of PTEN mutated compared to wild-type cells (Fig. 3). A similar effect was described in BRCA mutated breast cancer murine xenograft system, where despite a low efficacy of olaparib alone, there was a high efficacy of olaparib and BKM-120 in combination [59]. PTEN mutation, in prostate cancer was also associated to a higher sensitivity to a combined treatment with olaparib and BKM-120 [25].

In this study, we found that PTEN mutated cells have lower baseline levels of RAD51 protein and have a less pronounced RAD51 foci formation in response to DNA damage induced by doxorubicin compared to PTEN wild-type cells (Fig.4), These findings indicate that PTEN mutated cells had lower ability to repair double strand DNA breaks compared to the PTEN wild-type cell. However, we also observed some additive effect when combining olaparib and BKM-120 in PTEN wild-type cells (Fig. 3c). A possible explanation may be that PI3K inhibitors can create DSB defects, and can sensitize non-mutated PTEN cells to PARP inhibitors. The same observation has been published in BRCA competent triple negative breast cancer using BKM-120 and olaparib in mouse xenograft model [14]. The higher efficacy of PI3K-AKT-mTOR and PARP inhibitors beyond BRCA deficiency, using a double PI3K-mTOR inhibitor (GDC-0980) and olaparib have also been demonstrated in breast cancer [61].

The use of commercial cell lines limit the interpretation of our results since patient tumors are often more heterogeneous and the effects on cell lines are evaluated without the interactions of cancer cells with the in vivo microenvironment. The effective concentration of the inhibitors that are delivered to the cells can vary from in vitro to in vivo models, and thus results should be interpreted cautiously.

The rationale to investigate the clinical efficacy of dual PARP and PI3K inhibition in endometrial cancer should be further investigated using xenograft models at first. This approach could potentially expand the subset of patients who may benefit from PARP-inhibitors.

## Conclusion

Treatment options are limited for patients with metastatic or recurrent endometrial cancer. Here, we assessed the correlation between two novel therapeutics, PARP and PI3K-inhibitors, in the PTEN status of endometrial cancer cell lines. We found that PI3K-AKT-mTOR inhibition increases the efficacy of PARP inhibition in PTEN mutated cell lines which provides another argument in favor of an independent pathway linking PTEN to DNA repair and the HR complex. As the combined effect of PARP and PI3K inhibitors is synergistic in PTEN mutated cells, and to a lesser degree, in PTEN wild-type cells, it may also provide a therapeutic option for endometrial cancers not solely dependent on their PTEN status. In this preliminary study, BMN-673, a novel PARP inhibitor was found to be more efficacious compared to olaparib on selected endometrial cancer cell lines, Thus, this new PARP-inhibitor should be further tested in preclinical and later in clinical trials to evaluate its inhibitory efficacy compared to other PARP-inhibitors.

## Abbreviations

BRCA1/2: Breast cancer gene 1/2
CI: Combination index
DSB: Double strand break
HR: Homologous recombination
IC50: Inhibitory concentration
LVSI: Lymphovascular space invasion
mTOR: AKT-Mammalian Target of Rapamycine
PARP: Poly (ADP-ribose) polymerase
PTEN: Phosphatase Tensin homolog
SSB: Single strand break
